# Unveiling a novel S-Box strategy: The dynamic 3D scrambling approach

**DOI:** 10.1371/journal.pone.0329024

**Published:** 2025-09-02

**Authors:** Nayeem Ahmad Khan, Abdulbasid Banga, Taher M. Ghazal, Bayan Alabdullah, Nadeem Iqbal, Ahmad Alshamayleh, Atif Ikram, Hossam Diab

**Affiliations:** 1 Department of Information Technology, College of Computing and Informatics, Saudi Electronic University, Riyadh, Saudi Arabia; 2 College of Computing and Informatics (CCI), Saudi Electronic University, Riyadh, Saudi Arabia; 3 Hourani Center for Applied Scientific Research, Al-Ahliyya Amman University, Amman, Jordan; 4 Department of Information Systems, College of Computer and Information Sciences, Princess Nourah bint Abdulrahman University, Riyadh, Saudi Arabia; 5 Department of Computer Science and IT, The University of Lahore, Lahore, Pakistan; 6 Department of Data Science and Artificial Intelligence, Hourani Center for Applied Scientific Research, Al-Ahliyya Amman University, Amman, Jordan; 7 Faculty of Computer Science and Multimedia, Lincoln University College, Petaling Jaya, Malaysia; 8 Math and Computer Science Department, Faculty of Science, Menouï¬#129;a University, Menouï¬#129;a, Egypt; 9 Taibah University, Computer Science Department, Applied College, Madinah, Saudi Arabia; University of Lagos Faculty of Engineering, NIGERIA

## Abstract

A close study about the varied anatomies of the S-Box algorithms published in the literature indicates that no attempt has been made using the notion of 3D. To cement the more cryptographic security, this research article ventures to present a novel S-Box algorithm exploiting the notion of 3D. First of all, the algorithm initializes a 3D scrambled S-Box with the value of -1. After that, a 1D array is initialized with all the numbers of the potential S-Box. Now, the values placed in the 1D array are inserted randomly to the different cells of the 3D scrambled S-Box until all the numbers from the 1D array are not shifted. The way, the S-Box has been developed bears an ample promise to raise the inherent non-linearity of the required S-Box which, in turn, equips the S-Box to defy the potential cryptanalytic threats from the hackers and other adversaries. Lorenz chaotic system has been employed to produce streams of random numbers. Through rigorous analysis and experimentation, we demonstrate the efficacy of our approach in enhancing cryptographic security, offering robust protection against various cyber threats. Our research contributes to the advancement of S-Box design methodologies, providing a promising avenue for strengthening encryption algorithms in contemporary cryptographic systems. Potential real-world applications include secure communications in IoT devices, encryption in smart grid infrastructures, and data protection in medical imaging systems.

## 1 Introduction

In the realm of modern cryptography, design and implementation of robust encryption algorithms play a pivotal role in ensuring the security and confidentiality of sensitive information transmitted over digital networks. Cryptographers have shown growing interest in the critical issue of security, as evidenced by recent studies [1–7]. One fundamental component of many encryption schemes is the substitution box (S-Box), which is responsible for introducing nonlinear transformations to plaintext data, thereby enhancing the resistance of cryptographic systems against various attacks. The effectiveness of an S-Box hinges on its ability to introduce confusion and diffusion, two essential properties that thwart cryptanalysis attempts.

Traditionally, S-Boxes have been constructed using mathematical permutations and substitutions, with a focus on achieving high non-linearity and resistance against differential and linear cryptanalysis. However, as cryptographic standards evolve and computational capabilities advance, the need for more sophisticated and resilient S-Box designs becomes increasingly apparent. In the recent decade, cryptography experts have investigated many novel approaches for the construction of S-Boxes. These approaches span chaos theory [8], abstract algebra [9], Latin square [10] and even games [11]. Following are the few areas where S-Boxes are often embedded to boost the security measures.

- **Block ciphers**: S-Boxes have assumed much importance during the development of block ciphers. In these ciphers, they cause non-linearity, confusion and diffusion thus enhancing the security for the varied products. These boxes work on some fixed-size blocks of plaintext and render the corresponding ciphertext blocks with the equal size. Many block cipher algorithms have been developed in the past like DES, AES, Blowfish etc [12].
- **Stream ciphers**: Stream ciphers are an other security paradigm where the intrinsic potential of non-linearity being contained by the S-Boxes is exploited to encrypt or decrypt data streams continuously, bit by bit or byte by byte. Such ciphers are often deployed where data needs to be sent in some real time scenario like streaming, internet telephony of wireless communication [13].
- **Cryptographic hash functions**: This is yet another area where S-Boxes are deployed frequently. Cryptographic hash functions are part of many critical security products like digital signatures, password storage, and data integrity verification [14]. These functions fundamentally inject non-linearity and increase the avalanche effect, ensuring that small changes in input result in significant changes in the output hash value.
- **Message Authentication Codes (MACs)**: S-Boxes are also used in MAC algorithms to guarantee integrity and authenticity assurances for transmitted messages [15]. Besides, they are used to create a fixed-size tag from the message and a secret key. This tag is later on verified by the recipient using the same key.
- **Public key cryptography**: Although S-Boxes are not as prevalent in public key cryptography compared to its symmetric key counterpart, they are still employed in certain asymmetric encryption or signature schemes to boost security effects [22].
- **Digital signatures**: S-Boxes may play an important role while imparting security in the process of digital signatures. For instance, they may be used as a security component in the signing process in order to introduce non-linearity and randomness. In this way, it would become computationally infeasible for the hacker to forge a signature without having a knowledge of the secret key [17].
- **Secure multi-party computation**: A secure multi-party computation is an another area where the inherent potential of S-Box may be tapped [18]. Its deployment will ensure confidentiality and privacy when multiple parties compute some function jointly over their inputs while ensuring secrecy from each other.
- **Quantum cryptography**: In emerging domain of quantum cryptography, these S-Boxes hold potential for their applications. For instance, their deployment can enhance the security of quantum key distribution (QKD) protocols. These protocols utilize the principles of quantum mechanics to secure communication channels against eavesdropping and ensure the integrity of transmitted quantum information. S-Boxes may be employed to introduce non-linearity and confusion in quantum cryptographic schemes, contributing to the overall security of quantum communication networks [19].

In the last two decades, many S-Box algorithms have been developed. For instance, the work [20] wrote a novel algorithm to design 8 × 8 S-Boxes employing the constructs of discrete-space chaotic map and cuckoo search (CS) algorithm. The reported approach employed a 1D discrete-space chaotic map along with enormous key space for designing the required S-Boxes. By exploiting the inherent potential of improved artificial bee colony algorithm and discrete chaotic map, the study [21] designed chaotic S-Boxes. The peculiar modus operandi of the stated S-Box works like this. Firstly, the required S-Box was initialized through the opposition-based optimization. Secondly, in order to optimize the population, algorithm of transposition artificial bee colony (dual) was utilized. Besides, the Gaussian swap was engaged to cement the optimum efficiency. It is to be noted that opposition-based optimization performed self-learning for obtaining the better population. This happened due to the involvement of Gaussian mutation operator. The performance evaluation demonstrated that the suggested S-Box scheme rendered better results based on the varied evaluation metrics like bijection, strict avalanche criterion (SAC), non-linearity, bit independence criterion (BIC), I / O XOR distribution and linear approximation probability (LP).

In this article, we present a pioneering method for constructing a novel S-Box algorithm that harnesses the power of dynamic and unpredictable processes to enhance cryptographic security. Our approach leverages the principles of chaos theory and 3D scrambled approach to generate S-Boxes with superior non-linearity, confusion, and diffusion properties. By incorporating elements of randomness and unpredictability into the S-Box generation process, we aim to fortify encryption algorithms against emerging threats in the digital landscape.

The remainder of this article is organized as follows: in Sect 2, we provide an overview of the fundamental principles of S-Box design and the importance of non-linearity and diffusion in cryptographic systems. In Sect 3, we describe the Lorenz chaotic system which produces the stream of random numbers to carry out the logic of the suggested S-Box algorithm. Sect 4 presents our proposed methodology for generating novel S-Box algorithm based on chaotic system and the 3D scrambled approach. In Sect 5, we evaluate the performance and security of our approach through rigorous testing and analysis. A thorough discussion along with a comparison with other relevant studies has been made in the Sect 6. Conclusion and potential avenues for future exploration are addressed in the Sects 7 and 8.

## 2 Related work

A study of literature review about the security reveals that many S-Boxes have been developed in the past to impart security to the different products. For instance, the work [22] developed randomized S-Boxes which utilizes random-restart hill-climbing algorithm. Moreover, the reported work optimizes the inherent non-linearity of Boolean function with bijectivity constraints. One of the salient features of this new algorithm is that it reduced the computational overhead in a dramatic fashion. Apart from that, this algorithm struck a proper equilibrium among the three design principles of S-Boxes, i.e., speed of construction, dynamicity and cryptographic strength.

In an other study [23], a work has been presented for both the S-Box and the permutation box (P-Box). These twin purposes have been realized through the fusion of inversion map and symmetric group on Galois field. The reported method rendered a lot of non-linear substitution permutation boxes which of course, are furnished with the properties of diffusion as well as confusion. Apart from that, in the study [24], the notion of Rabinovich-Fabrikant (RF) system of coupled ordinary differential equations has been exploited for the construction of S-Box. As the modus operandi of the work, first of all, by sparking the said system of differential equations, random integers have been spawned. After that, these integers values have been scrambled to get the highly nonlinear chaotic S-Box. One of the main benefits of the designed algorithm is that, a just slight tempering in the initial values of the parameters of RF system can deliver an array of different cryptographically strong S-Boxes. Additionally, varied statistical and algebraic analyses have been carried out to gauge the performance of the novel S-Boxes. The authors of the reported work contend that the analyses contain very bright statistics which bear much importance of its potential applications in the domain of secure communications.

By synergizing the constructs of modular operations and heuristic evolution strategy, the work [25] came up with a novel algorithm for generating dynamic S-Boxes with high non-linearity. Besides, according to the contention of the authors of the reported work, plethora of robust S-Boxes can be developed just by a slight altering in the initial values of the proposed method. As the given algorithm was simulated, a specimen S-Box was generated. Moreover, its performance was evaluated against the state of the art benchmarks and criteria like bit independence criterion, differential uniformity, strict avalanche criterion, non-linearity, linear probability, and fixed points etc. Additionally, the S-Box was also employed in the encryption of digital images to demonstrate its significance in the real world problems. By spawning the pseudo-random sequences using the Gaussian distribution, the research [26] developed a novel algorithm for constructing dynamic S-Boxes. The authors of this work claim that their novel technique has overcome the intrinsic weakness of varied existing S-Boxes using the theory of chaos. In contrast to that, their work has leveraged the robustness of the pseudo-randomness sequences. Moreover, very low non-linearity was assessed in the proposed S-Boxes. This weakness has been addressed by using the genetic algorithms (GA) in order to maximize the non-linearity of Gaussian distribution-based S-Boxes. Lastly, the thorough security analysis of the given technique indicates that the proposed S-Boxes are equipped with ample promise for their application in the varied cyber security areas. Moreover, the work [27] came up with an S-Box algorithm through the usage of scaled Zhongtang chaotic system. Besides, the bits given by the random number generator were subjected to the NIST test series. All the tests were successfully passed. Additionally, the S-Box so written was tested based on the validation metrics like strict avalanche criteria, differential approximation probability, bit independence criteria, non-linearity, etc. Lastly, upon comparing the suggested S-Box with other published works, it was found that the suggested S-Box is furnished with more effective and stronger security. Moreover, the Table 1 shows some relevant works with their descriptions.

**Table 1:** Different S-Boxes with few parameters.

S-Box Technique	Chaotic map	Dimensions	Description
Ref. [28]	Hyperchaotic	4D	Galois field *GF*(2^8^) has been used
Ref. [29]	A novel chaotic map	1D	Development of a novel chaotic map using trigonometric functions
Ref. [30]	Rössler chaotic	3D	Fractional chaotic map has been employed. Non-linearity raised
Ref. [27]	Zhongtang chaotic	3D	An effective and strong S-Box design algorithm utilizing random number generator was developed
Ref. [31]	fractional-order (FO) Chen chaotic	3D	A simpler algorithm was given for the construction of S-box via time response of the FO chaotic Chen system
Ref. [32]	Hyperchaotic	5D	A novel method to construct cryptographically strong bijective substitution-boxes was developed
Ref. [11]	Hyperchaotic	5D	Chess piece Rook was used
Ref. [33]	Hyperchaotic	5D	A novel S-box design algorithm based on a new compound chaotic system

## 3 Lorenz chaotic system

Edward Lorenz in 1960 came up with a novel chaotic system dubbed as Lorenz system after his name. Nonlinear system of ordinary differential equations written below characterize this chaotic system. Apart from that, it is dynamical system [34]:

$$\begin{array}{c} \dot{x}=\sigma (y-x),\\ \dot{y}=x(\rho -z)-y,\\ \dot{z}=xy-\beta z \end{array}$$ (1)

**Fig 1:**
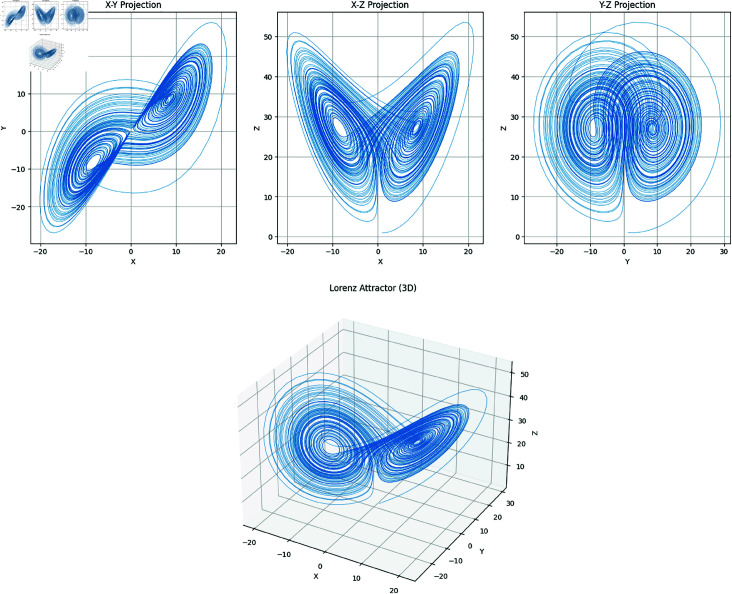
Lorenz attractors in 2D and 3D : (a) Attractors in three planes; (b) Attractors in 3D.

In the above set of equations, the variables *σ*, *ρ* and *β* correspond to the control parameters. Besides, state variables of this dynamical system are *x*,*y*,*z*. Using Runge–Kutta methods such as the RK45 [35], System (1) is commonly solved numerically. Additionally, other methods can also be applied. The chaotic attractors of both the 2D and 3D have been drawn in the Fig 1.

The Lorenz chaotic system was chosen due to its strong sensitivity to initial conditions, complex dynamic behavior, and proven unpredictability—qualities that are highly desirable in cryptographic applications. Compared to other chaotic maps, the Lorenz system offers a higher degree of randomness and a broader state space, which enhances the security of the generated keys [34]. Its continuous-time nature also enables more granular control in generating pseudo-random sequences. These attributes collectively contribute to the robustness of the proposed S-Box design.

**Fig 2:**
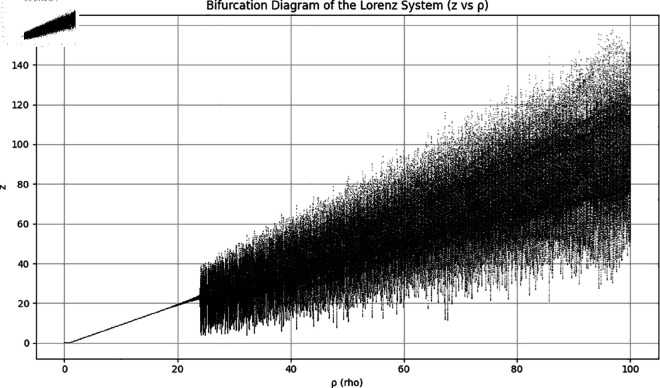
Bifurcation diagram of the Lorenz system.

**Fig 3:**
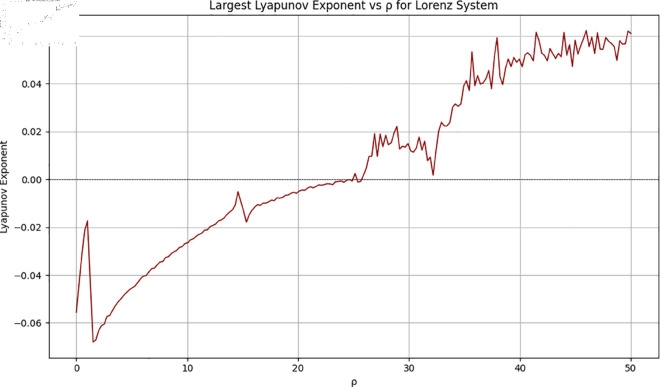
Lyapunov exponents of the Lorenz system.

Apart from that, the Figs 2 and 3 show the bifurcation diagram and the Lyapunov exponents of the reported chaotic system.

## 4 Proposed S-Box algorithm

As cryptographic threats evolve, the design of S-Boxes must continually advance to ensure robust security. Motivated by this need, our proposed methodology explores a novel approach based on three-dimensional (3D) structures.

The 3D structure introduces an additional layer of complexity in the substitution process, which significantly increases the non-linearity and confusion characteristics of the S-Box. Unlike traditional 2D S-Boxes that operate within a planar mapping framework, our 3D approach leverages spatial transformations across three axes, enabling a broader permutation space and more intricate scrambling of input-output relationships. This added dimensionality not only complicates the reverse engineering efforts of potential attackers but also helps resist various cryptanalytic attacks such as linear and differential cryptanalysis.

**Fig 4:**
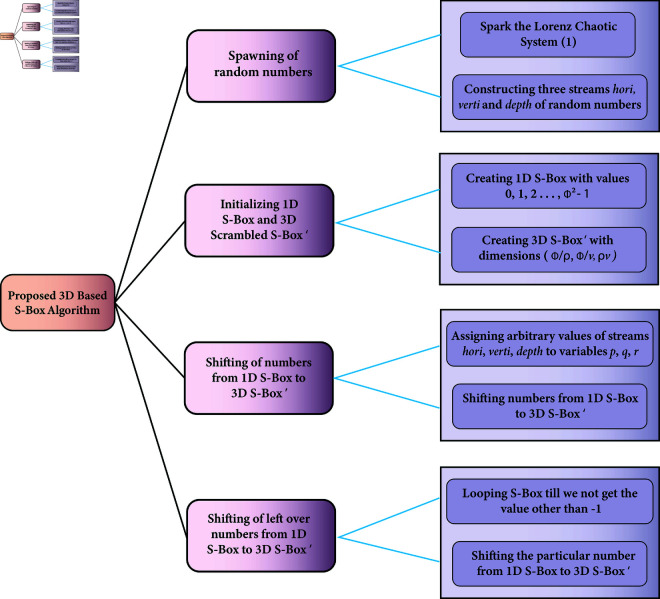
Proposed methodology.

The suggested S-Box algorithm has been developed for some arbitrary square dimensions say *ϕ* × *ϕ*. This algorithm has been developed upon the idea of dynamic 3D scrambled S-Box. First of all, a “raw” S-Box is created with the values of 0 to $\phi^{2}$ − 1. All the cells of this 3D box are initialized with the value of -1. Now, these values have been placed randomly over the arbitrary cells of the dynamic 3D scrambled S-Box. There are ample chances that some cells would have already been filled due to which a collision may occur so, a filter has been embedded to avoid such collision. After that, the remaining integers of the S-Box are inserted in the empty cells of 3D scrambled S-Box. Fig 4 shows these steps in a systematic fashion. Now, we will formally detail all the steps of the suggested algorithm.

Call the Algorithm 1 with the list of parameters *x*_0_, *y*_*0*_, *z*_*0*_, *σ*, *ρ*, *β*, *ϕ*, *υ*, *ψ*.

**Step 1:** As the Lorenz Chaotic System (1) is sparked, three arrays of random data, *i.e*., *x*, *y* and *z* are spawned. The arrays so obtained are not suitable for the particular logic of the algorithm, so they have been further processed to set of equations. In this way, three new arrays were obtained namely *hori*, *verti* and *depth* each of size $\phi^{2}$ (Lines 2 - 4).

**Step 2:** Line 5 creates a “raw” S-Box named as $\{S-Box_{k}{\}}_{k=0}^{\phi^{2}-1}$.

**Step 3:** Lines 6 - 12 initialize a 3D scrambled S-Box with the value of -1. Its name is *S* − $Box^{\prime }$ with the dimensions of $\frac{\phi }{\upsilon }$ × $\frac{\phi }{\psi }$ × υψ. We may observe here that the variables *υ* and *ψ* can assume any value unless they comply with mod(ϕ,υ)=0 and mod(ϕ,ψ)=0. In this way, we may have more than one dynamically created 3D scrambled S-Boxes. This act will enhance the security effects. If we multiply $\frac{\phi }{\upsilon }$, $\frac{\phi }{\psi }$ and υψ, same number of values for the original S-Box of size $\frac{\phi }{\upsilon }\times \frac{\phi }{\psi }\times \upsilon \psi =\phi^{2}$ will be obtained.

**Step 4:** Line 13 loops the index *iterator* which iterates from 1 to $\phi^{2}$. In each iteration, it assigns the ${\text{iterator}}^{\text{th}}$ values of streams *hori*, *verti* and *depth* to the temporary variables *p*, *q* and *r* respectively. Line 17 carries out a very critical task. If the *if* condition at the line 17 evaluates to be true, the ${\text{iterator}}^{\text{th}}$ value from the *S*–*Box* gets assigned to the scrambled *S* − $Box^{\prime }$ at the position (*p*,*q*,*r*). Line 19 assigns *S*–*Box* the value of -1 at the index *iteraotr* to keep track its used values.

**Fig 5:**
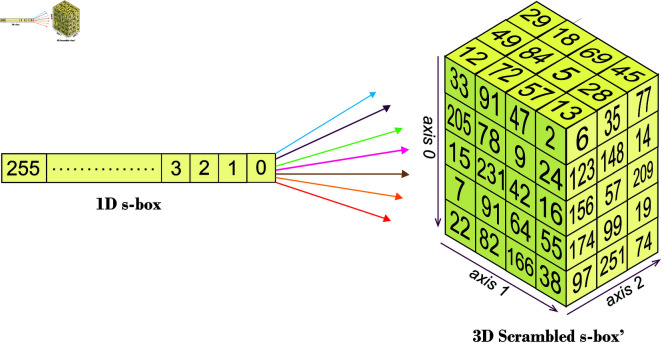
Shifting of integers from 1D s-box to 3D scrambled s-box’.

**Step 5:** As soon as the integers from the linear array *S*–*Box* get transferred to the 3D scrambled S-Box *S* − $Box^{\prime }$, the algorithm shifts the left over integers of *S* − $Box(1,\phi^{2})$ to the empty cells of *S* − $Box^{\prime }(\frac{\phi }{\rho },\frac{\phi }{\upsilon },\rho \upsilon )$ (Lines 22 - 35). Reshape this scrambled 3D S-Box *S* − $Box^{\prime }(\frac{\phi }{\upsilon },\frac{\phi }{\psi },\upsilon \psi )$ to *S* − $Box^{\prime }(\phi ,\phi )$ and return it. Fig 5 shows the shifting of integers from the 1D S-Box to its 3D scrambled counterpart in an intuitive way.

**Algorithm 1. Proposed 3D S-Box algorithm.**

**Input:** *x*_0_, *y*_0_, *z*_0_, *σ*, *ρ*, *β*, *ϕ*, *υ*, *ψ*

**Output:** *S* − $Box^{\prime }$

1: Iterate the chaotic system (1) to generate $\{x_{i}{\}}_{i=1}^{\phi^{2}+n_{0}}$,

$\{y_{i}{\}}_{i=1}^{\phi^{2}+n_{0}}$ and $\{z_{i}{\}}_{i=1}^{\phi^{2}+n_{0}}$. ⊳ Here $n_{0}\geq 500$. To avoid the

transient effect of the chaotic map, the first *n*_0_ values are

neglected.

2: $hori(i)\leftarrow floor(mod(abs(x(i+n_{0}))-floor(abs(x(i+n_{0})))\times 10^{14},\frac{\phi }{\upsilon }))+1$

⊳ Horizontal index calculation

3: $verti(i)\leftarrow floor(mod(abs(y(i+n_{0}))-floor(abs(y(i+n_{0})))\times 10^{14},\frac{\phi }{\psi }))+1$

⊳ Vertical index calculation

4: $depth(i)\leftarrow floor(mod(abs(z(i+n_{0}))-floor(abs(z(i+n_{0})))\times 10^{14},\upsilon \psi ))+1$

⊳ Depth index calculation

5: Set *S* − Box[k]←k+1 $\forall k\leftarrow 0,1,2,...,\phi^{2}-1$ ⊳ Set S–Box array

to the “raw” numbers

6: **for** p←1 to $\frac{\phi }{\upsilon }$ **do**

7:   **for** q←1 to $\frac{\phi }{\psi }$ **do**

8:    **for** r←1 to υψ **do**

9:     *S* − $Box^{\prime }(p,q,r)\leftarrow -1$ ⊳ Initialize 3D scrambled

*S* − $Box^{\prime }$ with -1

10:    **end for**

11:   **end for**

12: **end for**

13: **for** iterator←1 to $\phi^{2}$ **do**

14:   p←hori(iterator)

15:   q←verti(iterator)

16:   r←depth(iterator)

17:   **if** *S* − $Box^{\prime }(p,q,r)=-1$ **then**

18:     *S* − $Box^{\prime }(p,q,r)\leftarrow S-Box(iterator)$ ⊳ Shift the numbers

from S–Box to the 3D scrambled *S* − $Box^{\prime }$ at the position

(p,q,r)

19:    *S* − Box(iterator)←−1

20: **end if**

21: **end for**

22: pointer←0

23: **for** p←1 to $\frac{\phi }{\upsilon }$ **do**

24:   **for** q←1 to $\frac{\phi }{\psi }$ **do**

25:    **for** r←1 to υψ **do**

26:     **if** *S* − $Box^{\prime }(p,q,r)=-1$ **then**

27:      **while** (S−Box(pointer+1))=−1 **do**

28:       pointer←pointer+1

29:      **end while**

30:      *S* − $Box^{\prime }(p,q,r)\leftarrow S-Box(pointer+1)$ ⊳ Put remaining

values of S–Box to 3D scrambled *S* − $Box^{\prime }$

31:      pointer←pointer+1

32:     **end if**

33:    **end for**

34:   **end for**

35: **end for**

36: Reshape *S* − $Box^{\prime }$ to ϕ×ϕ and return *S* − $Box^{\prime }$

**Example 1.** In order to understand the proposed algorithm in a better way, here a toy example will be given.

Suppose we take the values as ϕ=6,υ=2 and ψ=3.

Suppose again that the arrays *hori*, *verti* and *depth* are filled as (Lines 2 - 4).

hori={1,2,2,1,1,1,2,1,2,1,1,2,2,1,1,2,1,1,2,2,1,2,1,2,1,1,2,2,2,1,1,2,2,1,2,1}

verti={5,2,4,1,6,3,6,3,3,4,1,5,2,6,3,4,4,2,3,2,6,2,6,2,4,1,5,2,6,3,5,2,4,3,5,6}

depth={3,2,2,1,1,3,2,1,3,3,1,2,2,1,3,2,1,1,3,2,3,2,3,2,1,1,3,2,2,3,1,2,2,3,2,1}

*S* − Box={1,2,3,4,5,6,7,8,9,10,11,12,13,14,15,16,17,18,19,20,21,22,23,24,25,26,27, 28,29,30, 31,32,33,34,35,36} (Line 5).

**Fig 6:**
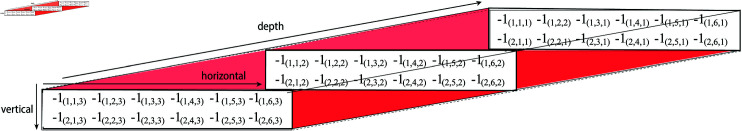
Initial state of a 2×6×3 S-Box.

As the lines 6-12 execute, the scrambled S-Box *S* − $Box^{\prime }$ is filled as shown in the Fig 6.

**Fig 7:**
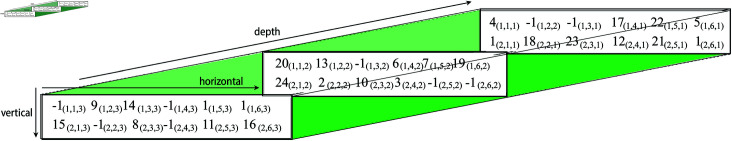
Intermediate state of a 2×6×3 S-Box.

After the execution of the lines 13-21, the partially scrambled S-Box *S* − $Box^{\prime }$ is filled as shown in the Fig 7.

**Fig 8:**
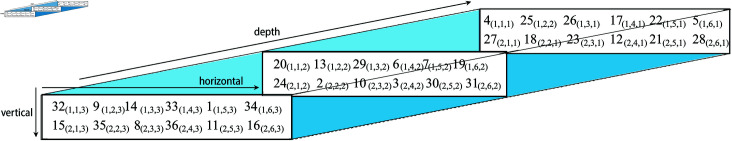
Final state of a 2×6×3 S-Box.

Next, the state of S-Box is shown in the Fig 8 after the execution of the lines 22–35. This is how scrambling is being realized in this study.

## 5 Simulation and security analyses

Upon calling the Algorithm 1 with the values {0.2895, 0.3051, 0.9277, 10, 28,8/3, 16, 8, 4}, the S-Box so generated can be viewed in the Table 2.

**Table 2:** Novel S-Box rendered by the suggested algorithm.

*i*/*j*	1	2	3	4	5	6	7	8	9	10	11	12	13	14	15	16
1	16	51	1	130	60	104	249	107	253	10	236	224	75	231	128	62
2	78	13	235	179	243	204	44	59	214	22	229	233	72	192	96	248
3	232	92	187	142	213	34	57	115	85	43	222	68	135	137	244	153
4	129	120	52	18	175	218	84	33	26	178	40	119	65	24	64	23
5	35	124	211	46	241	83	163	90	152	237	170	150	29	45	108	25
6	221	101	184	140	58	117	156	82	31	74	162	147	220	3	76	134
7	174	70	20	39	66	27	205	194	212	145	19	111	190	14	73	7
8	240	160	54	30	247	38	32	6	217	12	182	216	28	168	230	183
9	196	177	144	80	209	94	67	189	149	234	50	136	63	103	97	154
10	93	167	208	251	186	176	143	109	157	122	180	102	210	17	181	106
11	114	155	166	226	95	48	158	61	131	11	2	141	116	254	126	225
12	191	227	41	81	203	36	252	193	201	87	172	185	199	255	138	98
13	49	123	148	139	250	88	195	99	238	228	159	246	188	164	169	113
14	89	86	0	37	207	206	197	79	77	118	215	100	133	173	165	121
15	9	125	91	110	5	21	245	53	112	4	200	8	105	151	15	132
16	69	242	47	223	55	146	198	56	127	71	239	219	42	202	171	161

Merely creating cryptographic products is insufficient; they must undergo rigorous evaluation against the prevalent standards, criteria, and benchmarks established by security experts, cryptographers, and analysts. Here, we will showcase the resilience and effectiveness of suggested S-Box by subjecting it to such assessments. These include differential probability (DP), Strict Avalanche Criterion (SAC), bijectivity, linear probability (LP), non-linearity (NL), and Bit Independence Criterion (BIC) [36].

### 5.1 Bijectivity

In general, a *P* × *P* S-Box is considered bijective in character and orientation when it encompasses $2^{\text{P}}$ unique integers [37]. Moreover, these integers are confined in the range [0,2^*P*^–1]. Table 2 evidently satisfies bijectivity criterion.

### 5.2 Non-linearity

From time to time, hackers may attempt linear cryptanalysis attacks on security products [38]. To mitigate this risk, the developed S-Boxes must incorporate a significant degree of non-linearity. If a linear mapping exists between ciphertext and the corresponding plaintext, adversaries and other opponents can exploit the employed S-Box. To assess potential non-linearity of an *n*-bit Boolean function, denoted as *b*(*k*), mathematical equation written below (2) is normally utilized [39].

$$NL(b)=\frac{1}{2}\{2^{n}-max_{h\in \{0,1{\}}^{n}}|WS_{b}(h)|\}$$ (2)

In accordance to this equation, *WS*_*b*_(*h*) denotes the Walsh spectrum of a specified function *b*. Additionally, subsequent mathematical expression aids in computing non-linearity of an *n*-bit Boolean function *b*(*k*).

$$WS_{b(h)}=\sum_{x\in \{0,1{\}}^{n}}(-1{)}^{b(x)⊕h.x}$$ (3)

According to the above equation, $h\in \{0,1{\}}^{n}$. Additionally, *h*.*x* corresponds to dot product of *x* and *h*. The following gives more expressive form of this dot product

$$h.x=(h_{1}⊕x_{1})+...+(h_{n}⊕x_{n})$$ (4)

106, 106, 108, 108, 106, 108, 108, and 104 are non-linearity values for suggested S-Box. Additionally, the minimum, maximum, and average values are 104, 108, and 106.75, respectively. Furthermore, one can view all eight constituent Boolean functions giving the non-linearity values in Table 3.

**Table 3:** Non-linearities’ findings against the proposed S-Box.

Boolean functions	*f*_1_	*f*_2_	*f*_3_	*f*_4_	*f*_5_	*f*_6_	*f*_7_	*f*_8_
Non-linearity values	106	106	108	108	106	108	108	104

### 5.3 Strict Avalanche Criterion (SAC)

This benchmark states that upon changing only one input bit *n*, 0.5 probability ought to be there that ensuing resultant bit *m* will alter accordingly [39]. In simpler terms, if SAC result is approximated to 0.5, S-Box under consideration possesses an adequate level of randomness and chaotic behavior. Additionally, Table 4 displays the calculated results of the novel S-Box. Sometimes, it is dubbed as a dependence matrix. Moreover, average SAC result for suggested S-Box calculates to be 0.4989, satisfying established criterion.

**Table 4:** SAC’s findings for suggested S-Box.

*i*/*j*	1	2	3	4	5	6	7	8
1	0.4531	0.4688	0.5000	0.5125	0.5625	0.5312	0.5156	0.4688
2	0.5938	0.4844	0.4375	0.4375	0.5781	0.5156	0.5000	0.5000
3	0.4844	0.5156	0.5469	0.5156	0.4531	0.4531	0.5781	0.5156
4	0.4688	0.5000	0.5000	0.5312	0.4844	0.5000	0.5625	0.5000
5	0.4688	0.4844	0.5625	0.4844	0.4219	0.5000	0.3750	0.5000
6	0.4375	0.4844	0.5125	0.5625	0.5000	0.5012	0.4688	0.4844
7	0.5000	0.5312	0.4844	0.5156	0.5000	0.5156	0.5156	0.4844
8	0.5156	0.5156	0.4844	0.4531	0.4844	0.4688	0.5312	0.5156

### 5.4 Bit Independence Criterion (BIC)

This is a yet another security parameter used by the experts to judge the robustness of some S-Box. It checks whether a change in a specific input bit, denoted as *q*, leads to distinct changes in output bits *r* and *s* [39]. If such a separation of output bits occurs, the S-Box is deemed successful in its objective. To fulfill this criterion, the constituent Boolean functions of the S-Box must adhere to non-linearity conditions. The computation of mathematical expression $\left(T_{a}[p]⊕T_{b}[q]\right)$ − $\left(T_{a}[p]⊕T_{b}[p]\right)$ is conducted for all inputs of *p* which spans from 0 to 255 inclusive. In this expression, *T* represents the S-Box. It is to be noted that *q* and *p* differ by only one bit in the process of this evaluation.

Apart from that, the efficacy of some S-Box is gauged by finding average BIC-SAC values which has been found for the entire input values, with values near 0.5. This value indicates an optimal performance of the S-Boxes. Tables 5 and 6 delineate the benchmark employed for assessing SAC and non-linearity for Boolean functions within the suggested S-Box. Additionally, average non-linearity and SAC results for the suggested S-Box are 103.25 and 0.5064 in a respective fashion.

**Table 5:** BIC Non-linearity findings.

*i*/*j*	1	2	3	4	5	6	7	8
1	-	107	104	102	102	103	107	103
2	105	-	100	102	103	100	104	104
3	103	104	-	108	105	101	105	106
4	98	102	102	-	107	101	104	103
5	101	101	106	109	-	103	101	100
6	104	103	102	98	105	-	103	103
7	105	104	105	103	100	106	-	101
8	104	105	104	103	105	102	101	-

**Table 6:** BIC-SAC findings.

*i*/*j*	1	2	3	4	5	6	7	8
1	-	0.5049	0.5412	0.5287	0.4977	0.5072	0.5496	0.5090
2	0.4923	-	0.4829	0.4823	0.5018	0.4908	0.4623	0.5089
3	0.5167	0.5434	-	0.4876	0.5172	0.5187	0.5490	0.5318
4	0.5096	0.4512	0.4976	-	0.5187	0.4861	0.4698	0.4954
5	0.5192	0.5276	0.5176	0.5120	-	0.5304	0.5076	0.5489
6	0.4987	0.4812	0.4872	0.5087	0.5295	-	0.4698	0.4858
7	0.5098	0.5498	0.5053	0.5081	0.5393	0.5281	-	0.5022
8	0.5158	0.4765	0.4812	0.5028	0.4791	0.4976	0.4912	-

According to the study [40] which was done by Carlisle and Stafford , an S-Box satisfying non-linearity and SAC benchmarks is thought of as fulfilling the requisite BIC property. For suggested S-Box, results of 103.25 and 0.5064 portray an ostentatious weak linear relationship among output bits. Such statistics, no doubt, signals to the fulfillment of the BIC property.

### 5.5 Linear Probability (LP)

Intrinsic relationship between output and input of an S-Box is further assessed using concept of linear probability [41]. A lower LP value is preferable for a defiant and resilient S-Box. Eq (5) has been employed to find the LP value. A maximum LP value of 0.1190 for suggested S-Box signifies that the S-Box exhibits ample resilience against linear cryptanalytic attacks.

$$LP=max_{a_{z},b_{z}\neq 0}|\frac{\#\{z\in N|z.a_{z}=T(z).b_{z}\}}{2^{n}}-\frac{1}{2}|$$ (5)

In accordance to this equation, the variables *T*, *a*_*z*_, and *b*_*z*_ denote S-Box, input, and output masks, in a respective way. Besides, 0≤N≤255.

### 5.6 Differential Probability (DP)

Hackers and other adversaries employ this strategy to have an illegitimate access over the plaintext. In this particular, strategy, the plaintext is attempted to be recovered from some ciphertext by examining discrepancies between pairs of ciphertexts and their corresponding plaintexts [42]. By scrutinizing these discrepancies, potential hackers may gain access to secret key. As far as a robust S-Box is concerned, the value of this security parameter should be lower. Eq (6) is used to find the value of DP.

The accompanying Table 7 vividly demonstrates that the suggested S-Box exhibits the value of this metric as 11/256 = 0.0430. Such outcome indicates a powerful defiance to various assaults differential cryptanalysis. Eq (6) gives underlying calculation of the differential probability (*DP*).

**Table 7:** Suggested S-box DP table.

*i*/*j*	0	1	2	3	4	5	6	7	8	9	A	B	C	D	E	F
0	6	6	8	6	6	6	6	6	6	6	6	6	8	6	6	6
1	8	6	6	6	6	6	6	8	6	6	6	6	6	6	6	6
2	6	8	6	6	8	6	6	6	6	6	6	8	6	6	6	6
3	6	6	8	6	6	6	6	6	8	6	6	6	8	6	6	6
4	6	6	6	6	6	8	6	6	6	6	6	6	6	6	6	6
5	6	8	6	6	6	8	6	6	6	6	6	8	6	6	8	6
6	6	6	6	8	6	6	6	6	6	6	8	6	6	6	6	6
7	4	6	6	6	6	8	6	6	6	6	8	6	8	11	8	8
8	8	6	6	6	6	8	6	6	6	6	6	6	8	6	8	6
9	6	6	8	6	6	6	6	6	6	6	6	6	6	6	6	6
A	6	6	6	8	6	6	6	6	8	6	6	6	8	6	6	6
B	6	8	6	6	6	6	8	6	8	6	8	6	8	6	8	6
C	6	6	6	6	6	8	6	8	6	8	6	6	6	6	6	8
D	8	6	6	6	8	6	6	6	6	8	8	6	6	8	6	8
E	6	8	10	6	6	6	8	8	8	8	6	10	8	8	6	8
F	8	6	6	6	8	6	6	6	6	6	8	6	6	6	6	-

$$DP=max_{{△}_{z}\neq 0,{△}_{y}}|\frac{\#\{z\in N|T_{\left(z\right)}⊕T_{\left(z⊕△z\right)}=△y\}}{2^{n}}|$$ (6)

Within this equation, △z and △y denote respective input and output differentials.

### 5.7 Investigation into fixed, reverse fixed point and short cycles properties

Numerous S-Boxes have been proposed in the literature, some of which suffer from vulnerabilities related to fixed points and reverse fixed points [23]. A fixed point occurs when an input value maps to itself within the S-Box, making the output identical to the input—an undesirable property that can be exploited by adversaries. Conversely, a reverse fixed point arises when an input maps to its binary complement. For example, if the input is 10110101 (decimal 181) and the output is 01001010 (decimal 74), this represents a reverse fixed point.

It is crucial for cryptographic designers to ensure that their S-Boxes are free from both fixed and reverse fixed points, as these characteristics can significantly compromise the security of systems utilizing such S-Boxes [25]. In the present study, the proposed S-Box was thoroughly evaluated and found to have zero fixed points and zero reverse fixed points, as shown in Table 8. Furthermore, a comparative analysis with several state-of-the-art approaches have been carried out. The suggested work beats these studies [23,32,43–50].

**Table 8:** Fixed and reverse fixed points analysis.

S-Box	Fixed	Reverse fixed
technique	points	points
Ref. [23]	4	1
Ref. [43]	1	4
Ref. [44]	2	1
Ref. [45]	2	1
Ref. [32]	2	1
Ref. [46]	2	5
Ref. [47]	1	3
Ref. [48]	1	2
Ref. [49]	1	1
Ref. [50]	1	2
Ref. [33]	0	0
Ref. [8]	0	0
Proposed	0	0

The proposed S-box was analyzed for its cycle structure. The analysis revealed a diverse distribution of cycle lengths, with no significant presence of fixed points or short cycles [51]. Most cycles exhibited lengths greater than 4, indicating strong resistance to attacks that exploit short cycles in permutation-based structures.

### 5.8 Deployment of the proposed S-Box in image encryption frameworks

**Fig 9:**
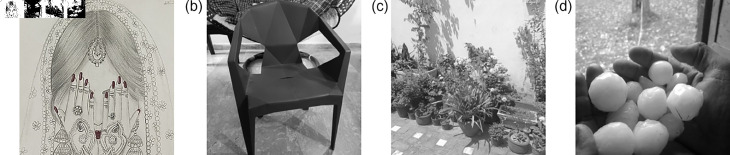
The plain input gray scale images: (a) Bride; (b); Chair (c) Flowers; (d) Hailstones.

**Fig 10:**
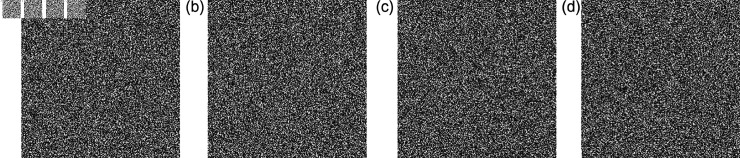
The cipher output gray scale images: (a) Bride; (b); Chair (c) Flowers; (d) Hailstones.

**Fig 11:**
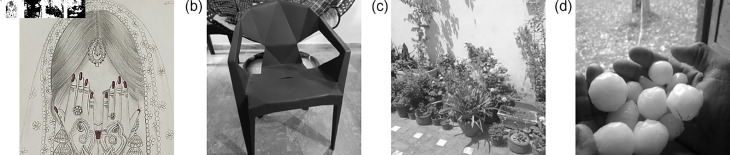
The decrypted gray scale images: (a) Bride; (b); Chair (c) Flowers; (d) Hailstones.

This section presents the application of the proposed 3D S-Box in the context of image encryption. The plain (Fig 9) encrypted (Fig 10) and subsequently decrypted images (Fig 11) are displayed to demonstrate the practical viability and effectiveness of the proposed method. As S-Boxes are a core component in introducing confusion during the encryption process, integrating the novel 3D S-Box enhances the security of image data by providing a higher degree of non-linearity and resistance to statistical and differential attacks. The results affirm that the proposed S-Box not only preserves visual integrity after decryption but also strengthens the encryption scheme against potential threats.

## 6 Discussion

This research introduces a novel algorithm for the construction of a 2D S-Box, uniquely enhanced by leveraging three-dimensional conceptualizations to inject non-linearity, a critical attribute for cryptographic strength. The promising results from this innovative approach underscore its potential to substantially improve cryptographic systems.

In the lines below, we give arguments in favor of the proposed work.

**Uniqueness of the 3D Approach**: The proposed S-box is indeed unique in its utilization of a 3D framework for its development. As highlighted in the manuscript, the literature review conducted revealed no prior instances of S-box construction based on a 3D notion. This innovative approach allows for enhanced complexity and non-linearity, which are critical factors in achieving high security in cryptographic systems.

**Theoretical Justification**: To further solidify the theoretical foundation of the proposed method, the proposed 3D S-box structure is furnished with the following characteristics.

- *Increased Confusion and Diffusion*: The 3D framework inherently introduces additional dimensions of permutation and substitution, leading to more complex transformations. This increased complexity enhances the confusion and diffusion properties of the S-box, making it more resistant to various cryptanalytic attacks.
- *Enhanced Avalanche Effect*: The introduction of the third dimension allows the proposed S-box to achieve a more significant avalanche effect, where a small change in the input leads to substantial changes in the output. This is a critical characteristic that strengthens the security of the cryptographic system.

Here, we again describe the particular modus operandi of the proposed algorithm. First of all, a “raw” 1D S-Box was created. After it, a 3D scrambled S-Box was created with all entries equal to -1. After that, a looping structure was introduced in the suggested algorithm which took elements from the 1D S-Box one by one and insert them to the different empty cells of the 3D S-Box. During this shifting from the 1D S-Box to the 3D scrambled S-Box, some of the numbers couldn’t be shifted. The reason of this stems from the fact that some cells were already filled. After it, one more looping structure was written in the proposed algorithm to shift the remaining integers of the 1D S-Box to the 3D scrambled S-Box. Lastly, the 3D S-Box was reshaped to the 2D S-Box to get the final result. 3D Lorenz chaotic system has been employed to spawn the three streams of random numbers. These random numbers played a cardinal role since they facilitated in locating a particular cell into which the numbers were shifted from the 1D S-Box to the 3D S-Box. To the best of our knowledge, no S-Box has been developed before it in which the notion of 3D has been employed.

When compared to conventional 2D S-Boxes, which are typically developed through direct two-dimensional methods, our S-Box stands out. The use of a 3D framework to guide the design process inherently integrates a higher degree of complexity and security. Metrics such as non-linearity scores and differential uniformity are significantly improved in our S-Box, demonstrating its superior ability to obscure the linear relationships that attackers often exploit.

The findings of the proposed work bears profound implications for the design of varied cryptographic systems. For instance, the real time implementation of the suggested S-Box can improve the security level of current encryption systems with minimum adaptation needed. One future avenue of this research may be to probe the scalability of the suggested method across different cryptographic standards and its defiance against emerging cryptanalytic techniques.

In summary, the incorporation of the notion of 3D for the development of a 2D S-Box represents a significant advancement in cryptographic design. It not only challenges traditional design paradigms but also sets a new benchmark for the integration of complex mathematical structures in practical encryption technologies. The superior performance of our 2D S-Box fortified by 3D conceptual methods marks a critical step forward in the ongoing evolution of cryptographic security.

Finally Table 9 presents a comparison of the outcomes from the proposed algorithm with results from other published studies. One can see that the non-linearity of the proposed work is better than majority of other published studies.

**Table 9:** A comparison of the performance of several S-boxes including the proposed one.

Study	Algorithm	NL	BIC-NL	SAC	BIC-SAC	LP	DP
Ref. [37]	Based on quantum-inspired QW and the customized PSO	107.00	103.0	0.5044	0.5066	0.1172	0.0313
Ref. [52]	Mackey–Glass equation	104.00	102.9	0.5000	0.4980	0.1328	0.0391
Ref. [10]	Enhanced logistic map and Latin square	105.25	103.2	0.5351	0.5000	–	0.0391
Ref. [53]	Jaya optimization algorithm	106.25	103.64	0.5009	0.4996	0.1171	0.0391
Ref. [11]	Rook and 5D chaotic system	106.125	103.17	0.5077	0.5061	0.1328	0.0469
Ref. [54]	3D chaotic map	106.00	104.2	0.4993	0.5030	0.1250	0.0391
Ref. [55]	Gingerbreadman chaotic system	102.00	102.9	0.5178	0.4999	0.1250	0.0313
Ref. [56]	logistic-sine map	105.25	103.8	0.4956	0.4996	0.1562	0.0391
Ref. [57]	quantum-inspired QW	106.00	103.9	0.4958	0.5023	0.1250	0.0313
Ref. [58]	Teaching-learning-based optimization	106.50	104.6	0.4995	0.4983	0.1172	0.0391
Ref. [29]	Chaotic map based on trigonometric functions	110	102.78	0.5001	-	0.1328	0.03906
Ref. [59]	Non-permutation binomial power functions	108	106.32	0.5001	-	0.1545	0.015625
Ref. [60]	Mordell Elliptic Curves over Galois Field	112	106.32	0.5032	0.5059	0.0625	0.0156
Ref. [61]	Chaotic map	104.25	104	0.5029	0.5059	0.127	0.0391
Suggested	3D Scrambled S-Box and Lorenz chaotic system	106.75	103.25	0.4989	0.5064	0.1190	0.0430

While the proposed S-box demonstrates strong cryptographic properties such as high nonlinearity, good avalanche behavior, and satisfactory resistance to linear and differential attacks, certain limitations remain. First, the S-box is statically defined, which may restrict adaptability in dynamic environments or key-dependent cipher designs. Second, the computational overhead of evaluating its cryptographic metrics could be high if extended to large-scale systems or embedded devices with limited resources. Lastly, the S-box has not yet been integrated or benchmarked within a complete cipher structure, which may affect the assessment of its practical real-world security performance.

## 7 Conclusion

S-Boxes enjoy a very central position in the realm of exciting domain of cyber security. These boxes are part and parcel of a lot of security products. This work has presented a novel S-Box which has been written using the notion of 3D. All the numbers of the required S-Box are inserted in the different 3D cells of the scrambled S-Box. This particular structure of the algorithm improves the non-linearity of the required S-Box. To the best of our knowledge, no S-Box has been written so far by using the 3D construct. To demonstrate the intrinsic defiance and resilience from the multifarious cyber security attacks, the suggested S-Box has been subjected to the different state of the art security parameters like differential probability (DP), Strict Avalanche Criterion (SAC), bijectivity, linear probability (LP), non-linearity (NL), and Bit Independence Criterion (BIC). The promising results of this study highlight the potential of the proposed algorithm to significantly advance cryptographic security, which in turn, has the bright prospects for its real world application.

## 8 Future work

Current research introduced a new S-Box algorithm aimed at enhancing varied cyber security products. There are several promising vistas for deeper investigation and refinement of this work. For example, 1) *IoT Security*: Given the implications of ubiquitous nature Internet of Things (IoT) devices, securing the communication and data exchange between these devices is crucial. The suggested S-Box algorithm of this work can be deployed in various IoT settings to cement the security protocols, ensuring the confidentiality and integrity of data transmitted between IoT devices. This can be realized by integrating the S-Box algorithm into IoT communication protocols or embedding it directly into IoT devices to encrypt precious and sensitive data. 2) *Cloud Computing Security*: No doubt, virtually all the major organizations have shifted their operations based on the cloud computing. But plethora of security concerns are a source of permanent threat to it. As we deploy the suggested S-Box diverse cloud computing platforms, data stored in the cloud can be encrypted with heightened security measures. This would, of course, bolster the protection of precious data stored in cloud servers, thus saving it from unauthorized access or breaches. 3) *Blockchain Technology*: This is yet another niche which is known for its decentralized and tamper-proof character. Ensuring the security of transactions and data on blockchains is a paramount concern. Integrating the suggested S-Box algorithm into blockchain networks has the potential to enhance the encryption mechanisms used to secure transactions and data blocks. This would strengthen the overall security of blockchain networks, thus mitigating the risk of hacking or data manipulation. 4) *Mobile Device Security*: With the ubiquitous use of tablets and smartphones, ensuring the security of mobile devices and the data, is essential. By incorporating the developed S-Box algorithm into mobile device operating systems or security applications, data stored on these devices can be encrypted with advanced cryptographic techniques. This would enhance the protection of sensitive information stored on mobile devices, guarding against unauthorized access or data breaches.
